# Four-stage Lobatto analysis on 3D magneto-hydrodynamic radiative non-newtonian nanofluid flow and heat transportation over a stretchable surface under Brownian motion and thermophorsis impact

**DOI:** 10.1016/j.mex.2025.103292

**Published:** 2025-03-28

**Authors:** Aaqib Majeed, Parvez Ali, Marouan Kouki

**Affiliations:** a Department of Mathematics, The University of Faisalabad, Sargodha Road, University Town, Faisalabad, 38000, Pakistan; b Department of Mechanical Engineering, College of Engineering, Qassim University, Buraydah, 51452, Saudi Arabia; c Department of Information System, Faculty of Computing and Information Technology, Northern Border University, Rafha, Saudi Arabia

**Keywords:** MHD, Three dimensional, Eyring-Powell nanofluid, Brownian motion, Viscous dissipation, Radiation, Algorithms, MATLAB, Four-Stage Lobatto Scheme with Finite Difference Method

## Abstract

Viscous dissipation and thermal radiation are essential in governing the dynamics of boundary layer flow, particularly in high-temperature engineering systems such as gas turbines, combustion engines, and industrial furnaces. Thermal radiation emerges as a primary factor of heat transfer under such conditions, while viscous dissipation contributes to the transformation of kinetic energy into thermal energy through internal frictional forces within the fluid. The present study investigates the three dimensional magneto-hydrodynamic (MHD) flow of a radiative Eyring-Powell nanofluid towards a stretchable, porous surface. The governing equations, initially formulated as partial differential equations (PDEs), are reduced to a set of coupled ordinary differential equations (ODEs) through similarity transformations. These transformed equations are numerically solved using MATLAB bvp5c solver. A detailed parametric analysis is performed to examine the impact of key dimensionless quantities, including slip parameter, Lewis number, Eckert number, thermophoresis parameter, magnetic field strength and Brownian motion parameter, on velocity profile, temperature profile and concentration distributions. The analysis reveals that the fluid velocity decreases with an increase in the magnetic field strength, whereas it exhibits an increasing trend with higher values of the Eyring–Powell fluid parameter. This paper convers the following key points:

• Modeled the dynamical flow equation for hybrid Eyring-Powell nanofluid.
• Analyzed the magnetic force impact on the velocity curve.
• Graphical interpretations are presented, highlighting the effects of physical parameters.

## Graphical abstract

**Figure:**
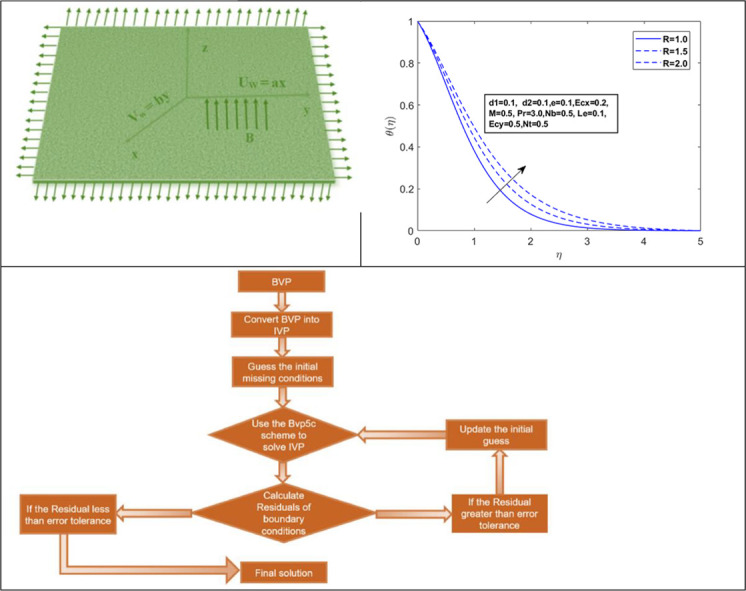

Specifications table

**Table:** 

Subject area:	Engineering
More specific subject area:	Boundary layer flow
Name of your method:	Four-Stage Lobatto Scheme with Finite Difference Method
Name and reference of original method:	Alotaibi H, Rafique K. Numerical simulation of nanofluid flow between two parallel disks using 4-stage Lobatto III-A formula. Open Physics. 2022 Jul 18;20(1):649–56.
Resource availability:	MATLAB

## Background

The unique properties of physiological fluids have led to their widespread application in various industrial processes, sparking a growing interest in non-Newtonian fluid models within the field of fluid mechanics. Over time, the research landscape has increasingly shifted towards non-Newtonian formulations due to their ability to capture complex fluid behaviors. Among these, models such as Carreau fluid, Prandtl-Eyring fluid, Eyring-Powell fluid, Eyring-Philippoff fluid, micropolar fluid, and Casson fluid, have gained significant prominence.

The Eyring-Powell model, in particular, has demonstrated remarkable adaptability across different stress regimes. Powell et al. [1] highlighted the model's exponential sensitivity to pressure at low-stress levels, transitioning to a linear relationship at higher stresses. Patel et al. [2] observed that the Eyring-Powell model outperformed the power-law model in certain applications. Sirohi et al. [3] examined the behavior of Eyring-Powell fluids near a dynamic plate using three specialized methods. Yoon et al. [4] clarified the distinct viscosities of this fluid model under small and large shear rates. Further advancements include Nadeem et al. [5], who analyzed peristaltic flow using the Eyring-Powell model, revealing that an increase in the Eyring-Powell flow parameter resulted in an expanded peristaltic pumping zone. Motsa et al. [6] investigated the incompressible boundary layer flow of Eyring-Powell nanofluids past a shrinking sheet, emphasizing the time-independent characteristics of this model. These studies underscore the versatility and growing relevance of non-Newtonian fluid model which addressing complex fluid dynamics challenges across various domains. Jaya Chandra et al. [7] and Hayat et al. [8] explored the implications and applications of stratification in non-Newtonian fluid model. The rapid progressions in nanotechnology, particularly in the medical and industrial fields, have further spurred interest in artificial nanofluids due to their unique thermal and rheological properties. The pioneering work by Choi et al. [9], which introduced nanoparticles into base fluids, laid the foundation for developing viscoelastic nanofluids with diverse applications. Experimental studies have demonstrated significant improvements in the thermal conductivity with the addition of tiny particles. This enhancement, often described as a hallmark of viscous nanofluids, has been extensively documented in the literature. For instance, Kuznetsov et al. [10] investigated the flow of viscous nanofluids along vertical plates, focusing on boundary layer phenomena. Similarly, Khan et al. [11] formulated the governing equations for non-Darcy laminar viscous flow past a stretching sheet embedded with nanoparticles (NPCs). Their work captured critical boundary conditions involving suction and blowing, providing insights into the dynamics of viscous microliquids under such conditions. These studies collectively highlight the transformative potential of nanofluids in enhancing heat and mass transfer, with significant implications for cutting-edge applications in both scientific and engineering domains. Reddy and Mangamma [12] employed numerical interactions between heat radiation and viscous dissipation on the magnetic Fe3O4-ethylene glycol flow of nanofluid over a shrinking surface with heat production and velocity slip impact. Yanala et al. [13] examined unsteady non-Newtonian nanofluid flow over a non-linear stretchable Riga plate under the influence of thermal radiation and chemical reaction.

Nadeem et al. [14] analyzed the laminar nano liquid flow considering suctions/injection effect published the breakthrough on the viscous micro liquid flow due to stretchable surface & carried out the computational techniques on nanofluid flow over stretching surface on the impact of heat transfer by Mabood et al. [13]. On the moving stretched vertical sheet Mabood et al. [15] analyzed the micro liquids flow towards exponential pressure dependent with convective type boundary. Majeed et al. [16] discussed the enhanced energy efficiency by considering magnetic field and heat transportation of hybrid nanofluid with variable viscosity. Shankar et al. [17] investigated the characteristics of Joule heating and thermal stratification on MHD nanofluid flow over an exponentially stretchable surface. Goud et al. [18] performed theoretically mixed convective viscous dissipative flow towards an infinite plate under porous medium and Soret impact. Mansur et al. [19] validated their research by demonstrating dual solutions under specific conditions. Malavandi et al. [20] numerically addressed the problem of nanofluid flow generated due to stretching at stagnation points and slip flow behavior of viscous, unstable fluids over stretching surfaces of theoretical dimensions. Similarly, Nazar et al. [21] expanded on these findings with additional formulations. Goud et al. [22] discussed Soret, Dufour effects on heat and mass transportation of magneto-Casson fluid towards a permeable vertical plate. They performed the numerical simulation by utilizing finite element method. Asogwa et al. [23] demonstrated the dynamics of suction impact on EMHD on stagnation point flow of Casson nanofluid over a stretchable Riga surface by considering MATLAB package. Ibrahim and Shankar et al. [24] provided critical insights behavior of nanofluid, specifically analyzing linear sub problems. Their study examined the velocity profiles of such flows, contributing to the understanding of nanofluid dynamics in advanced applications. The integration of magnetohydrodynamic (MHD) techniques with knowledge of nanomaterials and nanofluids has enabled their use in modern applications, such as wound therapy, optical modular motors, and power-based systems. Reddy and Goud [25] explored the steady electrically conducting MHD stagnation point flow over a stretching sheet. They also discussed the influence of convective boundary condition and thermal radiation by applying numerical scheme. Shoaib et al. [26] extended this research by investigating temperature field distributions and inequalities within 2D-3D domains, particularly over soil mediums, further highlighting the versatility of these advanced fluid systems in practical and theoretical contexts. Shehzad et al. [27] focused on creating a model for hybrid nanoliquid in permeable surface with MHD thermal transfer. Ghalambaz et al. [28] developed a model to describe heat transfer behavior within nanomaterials. Aly et al. [28] addressed the nonlinear expressions governing heat flux scenarios, building on insights from prior studies [[29], [30], [31], [32], [33], [34], [35], [36], [37], [38]]. These investigations include the magnetohydrodynamic (MHD) flow of Casson nanofluids, significantly enhancing our understanding of nanofluid dynamics and their diverse applications in engineering and industrial processes. This research aims to address the following question by its conclusion

• How does the viscous dissipation influence the flow characteristics of 3D nanofluid?
• What is the dynamical impact of Brownian motion and permeability parameters on boundary layer flow phenomena?
• How does the concentration profile affect the overall flow patterns and transport phenomena of the nanofluid?
• What are the combined influences of thermal radiation and non-Newtonian fluid parameters on the flow behavior and heat transfer characteristics in the system.

### Objective and novelty of the work

The primary objective of this research is to explore the dynamic behavior of fluid flow under the influence of the Eyring-Powell fluid model. The study delves into the effects of magnetic force on fluid velocity, offering a detailed analysis of convergence flow parameters through graphical visualizations. A significant aspect of this work is the inclusion of the velocity ratio parameter, which provides deeper insights into the underlying flow dynamics. Furthermore, the study meticulously accounts for viscous dissipation, emphasizing its impact on both thermal and fluid properties. Employing a robust numerical approach, the model is solved using MATLAB to ensure the generation of precise, reliable, and meaningful results. This comprehensive approach fills a critical gap in understanding complex fluid dynamics, particularly for non-Newtonian fluids, providing new perspectives and valuable insights for theoretical and practical applications.

## Problem description

**Fig. 1:**
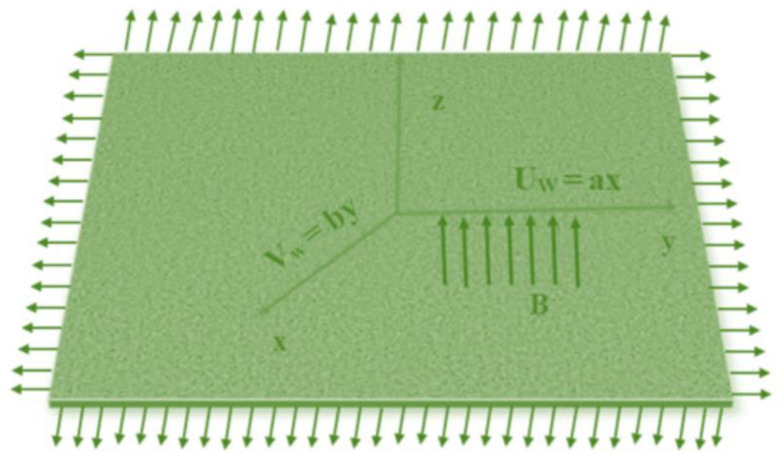
Physical representation of the model.

Here we considered viscous, incompressible, and electrically conductive fluid over a surface. A coordinate axes unit is used, and origin is placed at the position “O” and the surface positioned along the surface at z=0, where the flow in the directed into region z>0. At the fixed origin, the velocity components along the x and y axes are represented by $U_{w}\left(x\right)\;=\;ax$ and $V_{w}\left(x\right)\;=\;by$, respectively, where a and b are positive constants, as depicted in Fig. 1. The nanoparticle temperature and concentration at the surface of the sheet are denoted by $T_{f}$ and $C_{w}$ represent the ambient fluid temperature and concentration. Hall current effects are disregarded, and it is assumed that the Reynolds number is significantly smaller compared to the influence of the external magnetic field. Additionally, the Cauchy stress tensor T is appropriately defined for the current non-Newtonian fluid model.

(1) T=−ρl+τ,

(2) $$\rho_{f}a_{i}=-\nabla \rho +\nabla \;.\left(\tau_{ij}\right)+\sigma \;\left(J\;\times \;B\right).$$

In this context we use symbols like p to represent pressure, I to specify the identity tensor, σ is the fluid conductivity, and the stress tensor based on the Eyring Powell fluid model. The equation, for $\tau_{ij}$ can be expressed as $\tau_{ij}$ = $\mu \left(\partial u_{i}/\partial u_{j}\right)\;+\;\left(1/\beta \right)sinh^{-1}[\left(1/\gamma \right)\left(\left(\partial u_{i}/\partial x_{j}\right)\right]$ where βandγ denote key features of the Eyring Powell fluid and μ stands for coefficient of viscosity as follows:

(3) $$sinh^{-1}\left(\frac{1}{\gamma }\frac{\partial u_{i}}{\partial x_{j}}\right)≅\frac{1}{\gamma }\frac{\partial u_{i}}{\partial x_{j}}-\frac{1}{6}\left(\frac{1}{\gamma }\frac{\partial u_{i}}{\partial x_{j}}\right)\;\&\;\left|\frac{1}{\gamma }\frac{\partial u_{i}}{\partial x_{j}}\right|<1,$$

Gireesha et al. (28) gave the equations controlling mass, energy, momentum, and the volume percentage of nanoparticles for the Eyring-Powell nanofluid. This aligns with the made assumptions.

### Governing equations

The governing equations for the problem consist of the continuity equation, momentum equations, energy equation, and concentration equation, which describe the behavior of the flow, heat transfer, and mass diffusion in the system. These equations incorporate the effects of the Eyring-Powell fluid model, magnetic fields, and thermal considerations [30,31]:

Continuity Equation

(4) $$\frac{\partial u}{\partial x}\;+\;\frac{\partial v}{\partial y}\;+\;\frac{\partial w}{\partial z}\;=0,$$

x-Momentum Equation:

(5) $$u\frac{\partial u}{\partial x}\;+v\;\frac{\partial u}{\partial y}+w\frac{\partial u}{\partial z}\;=\nu \;\frac{\partial^{2}u}{\partial z^{2}}\;+\;\frac{\mu }{\beta \gamma \rho_{f}^{2}}\frac{\partial^{2}u}{\partial x^{2}}\;-\;\frac{1}{\beta \gamma^{3}\rho_{f}}{\left(\frac{\partial u}{\partial z}\right)}^{2}\frac{\partial^{2}u}{\partial z^{2}}-\frac{\sigma B_{0}^{2}}{\rho_{f}},$$

y-Momentum Equation:

(6) $$u\;\frac{\partial v}{\partial x}+v\;\frac{\partial v}{\partial y}+w\frac{\partial v}{\partial z}=\nu \;\frac{\partial^{2}v}{\partial z^{2}}+\frac{\mu }{\beta \gamma \rho_{f}^{2}}\frac{\partial^{2}v}{\partial x^{2}}-\frac{1}{\beta \gamma^{3}\rho_{f}}{\left(\frac{\partial v}{\partial z}\right)}^{2}\frac{\partial^{2}v}{\partial z^{2}}\;-\frac{\sigma B_{0}^{2}}{\rho_{f}}v,$$

Energy Equation

(7) $$u\;\frac{\partial T}{\partial x}+v\;\frac{\partial T}{\partial y}+w\frac{\partial T}{\partial z}=\alpha_{m}\frac{\partial^{2}T}{\partial z^{2}}-\frac{1}{{\left(\rho c_{p}\right)}_{f}}\frac{\partial q_{r}}{\partial z}+\tau \left(D_{B}\left(\frac{\partial C}{\partial z}\frac{\partial T}{\partial z}\right)+\frac{D_{T}}{T_{\infty }}{\left(\frac{\partial T}{\partial z}\right)}^{2}\right)+\frac{\sigma B_{0}^{2}}{{\left(\rho c_{p}\right)}_{f}}\left(u^{2}+v^{2}\right)+\frac{\mu }{{\left(\rho c_{p}\right)}_{f}}\left\{{\left(\frac{\partial u}{\partial z}\right)}^{2}+{\left(\frac{\partial v}{\partial z}\right)}^{2}\right\},$$

Concentration Equation

(8) $$u\;\frac{\partial C}{\partial x}+v\;\frac{\partial C}{\partial y}+w\frac{\partial C}{\partial z}=D_{B}\frac{\partial^{2}C}{\partial z^{2}}+\frac{D_{T}}{T_{\infty }}\frac{\partial^{2}T}{\partial z^{2}}.$$

To sum up, the velocities in the directions of x,y,andz are represented by the letters u,vandw respectively. The constants TandC stand for the fluid's temperature and species concentration respectively. Kinematic viscosity (v), thermal diffusivity $\alpha_{m}=\kappa /{\left(\rho c_{p}\right)}_{f}$ thermal conductivity (k), Brownian diffusion coefficient ($D_{B}$), liquid density $\left(\rho_{f}\right)$, magnetic field strength ($B_{0\;}$), thermophoretic diffusion coefficient ($D_{T}$) and the ratio of nanoparticles productive heat capacity to that of a regular fluid $\tau ={\left(\rho c_{p}\right)}_{p}/{\left(\rho c_{p}\right)}_{f}$ are additional significant parameters. Furthermore, the heat measurements of the conventional fluid and nanoparticles are denoted by ${\left(\rho c_{p}\right)}_{f}$ and ${\left(\rho c_{p}\right)}_{p}$ respectively.

(9) $$q_{r}=\frac{-4\sigma^{*}}{3k_{1}}\frac{\partial T^{4}}{\partial z}.$$

Here $\;k_{1},\sigma^{*}$ indicates absorption coefficient and Stefan–Boltzmann constant. Using$\;q_{r}$ in Eq. (9), we can get

(10) $$\begin{array}{ccc} u\;\frac{\partial T}{\partial x}+v\;\frac{\partial T}{\partial y}+w\frac{\partial T}{\partial z} & = & \left(\alpha_{m}+\frac{16\;\sigma^{*}T_{\infty }^{3}}{3{\left(\rho c_{p}\right)}_{f}k_{1}}\right)\frac{\partial^{2}T}{\partial z^{2}}+\tau \left(D_{B}\left(\frac{\partial C}{\partial z}\frac{\partial T}{\partial z}\right)+\frac{D_{T}}{T_{\infty }}{\left(\frac{\partial T}{\partial z}\right)}^{2}\right)+\frac{\sigma B_{0}^{2}}{{\left(\rho c_{p}\right)}_{f}}\left(u^{2}+v^{2}\right)\\ & & +\;\frac{\mu }{{\left(\rho c_{p}\right)}_{f}}\left\{{\left(\frac{\partial u}{\partial z}\right)}^{2}+{\left(\frac{\partial v}{\partial z}\right)}^{2}\right\}, \end{array}$$

The relevant boundary constraints, Dhlamini et al. [29], Sreedevi et al. [31], and Nayak et al. [30] for the flow problem are:

(11) $$\begin{array}{ccc} & & u=U_{w}\left(x\right),v=V_{w}\left(y\right),w=0,\;T=T_{w},C=C_{w}\;\text{at}\;z=0\\ & & u\to 0,\;v\to 0,\;T\to T_{\infty },\;C\to C_{\infty }asz\to \infty . \end{array}$$

Where, $h_{f}$ state heat transfer coefficient. Similarity transformations, as was introduced by Chamkha and Khaled et al. (32), convert PDEs to ordinary differential equations, thereby further decreasing the mathematical representation of the problem:

### Scaling transformation

Similarity variable used for the current problem are:

(12) $$\begin{array}{ccc} & & u=axf^{\prime }\left(\eta \right),v=ayg^{\prime }\left(\eta \right),\omega =-\sqrt{av}\left(f\left(\eta \right)+g\left(\eta \right)\right)\\ & & \theta \left(\eta \right)=\frac{T-T_{\infty }}{T_{f}-T_{\infty }},\left(\eta \right)=\frac{C-C_{\infty }}{C_{w}-C_{\infty }},\eta =\sqrt{\frac{U_{w}}{vx}}z \end{array}$$

Ordinary Differential Equation:

By utilizing the similarity approach reduce the Eqs. (4) to (10) in the form of ODEs are as follows:

(13) $$\left(1+\varepsilon \right)f^{\prime \prime \prime }\;+\left(f+g\right)f\;-\left(f\;\right)2-ed_{1}\left(f\;\right)2f\;-Mf\;=0,$$

(14) $$\left(1+\varepsilon \right)g^{\prime \prime \prime }+\left(f+g\right)g^{\prime \prime }-{\left(g^{\prime }\right)}^{2}-ed_{2}{\left(g^{\prime \prime }\right)}^{2}g^{\prime \prime \prime }-Mg^{\prime }=0,$$

(15) $$\left(1+\frac{4}{3}R\right)\theta^{\prime \prime }+Pr\left(f+g\right)\theta^{\prime }+PrN_{b}\phi^{\prime }\theta^{\prime }+PrN_{t}{\left(\theta^{\prime }\right)}^{2}+B_{1}\theta +Ec_{x}Pr\left({\left(f^{\prime \prime }\right)}^{2}+M{\left(f^{\prime }\right)}^{2}\right)+Ec_{y}Pr({\left(g^{\prime \prime }\right)}^{2}+M{\left(g^{\prime }\right)}^{2}=0,$$

(16) $$\phi^{\prime \prime }+LePr\left(f+g\right)\phi^{\prime }+\frac{N_{t}}{N_{b}}\theta^{\prime \prime }=0.$$

### Boundary conditions

The relevant boundary principles are outlined as follows:

(17) $$at\;\eta =0,f\left(\eta \right)=0,g\left(\eta \right)=0,f^{\prime }\left(0\right)=1,\;g^{\prime }\left(0\right)=A,\;\theta \left(0\right)=1,\phi \left(0\right)=1\;and\;as\;\eta \to \infty ,f^{\prime }\left(\eta \right)\to 0,g^{\prime }\left(\eta \right)\to 0,\theta \left(\eta \right)\to 0,\phi \left(\eta \right)\to 0,$$

Where

(18) $$\begin{array}{ccc} d_{1} & = & \frac{a^{3}x^{2}}{2\nu \gamma^{2}},\;N_{t}=\left[\tau D_{T}\left(T_{f}-T_{\infty }\right)\right]/T_{\infty }\nu \\ d_{2} & = & \frac{b^{3}y^{2}}{2\nu \gamma^{2}},\;R=4\sigma^{*}T_{\infty }^{3}/\rho_{f}C_{f}\alpha_{m}k_{1}\\ e & = & \frac{1}{\mu \beta \gamma },\;N_{b}=\left[\tau D_{B}\left(C_{w}-C_{\infty }\right)\right]/\nu \\ M & = & \frac{\sigma B_{0}^{2}}{\rho_{f}a},\;A=\frac{b}{a}\\ Pr & = & \frac{\nu }{\alpha_{m}},\;Le=\frac{\alpha_{m}}{D_{B}}\\ Ec_{x} & = & \frac{U_{w}^{2}}{\left[c_{p}\left(T_{f}-T_{\infty }\right)\right]},\;Ec_{y}=\frac{V_{w}^{2}}{\left[c_{p}\left(T_{f}-T_{\infty }\right)\right]} \end{array}$$

Also note that when $e=d_{1}=d_{2}=0,$ The current model reduce to Newton fluid.

## Skin friction, Nusselt number and Sherwood number

The quantity of practical concern $\left(C_{f_{x}}and\;Nu\right)$ are stated below

(19) $$C_{f_{x}}=\frac{\tau_{w}}{\rho_{f}U_{w}^{2}},Nu=\frac{xq_{w}}{\kappa \left(T_{f}-T_{\infty }\right)},$$

Here, $q_{w}$ and $\tau_{w}$ represents wall flux and shear stress on surface. Using above transformation, we get

(20) $$\left(1/\sqrt{Re_{x}}\right)Nu=-\theta^{\prime }\left(0\right),$$

(21) $$\frac{Sh_{x}}{\sqrt{Re_{x}}}=\;-\phi^{\prime }\left(0\right),$$

(22) $$\left(1/\sqrt{Re_{x}}\right)C_{f_{x}}=-f^{\prime \prime }\left(0\right).$$

Where $Re_{x}=U_{w}x/\nu$ is the local Reynolds number.

## Method details

### Detailed explanation of bvp5c, Lobatto formula, and collocation method

The bvp5c function in MATLAB is a robust and widely used solver for boundary value problems (BVPs) for ordinary differential equations (ODEs). It is particularly designed to handle two-point boundary value problems by using a collocation approach based on the Lobatto formula, coupled with automatic mesh refinement and error control mechanisms**.**

### Lobatto formula

The Lobatto formula is a type of implicit Runge-Kutta method that uses specific collocation points called Lobatto points. These points include both endpoints of the interval, typically 0 and 1, and additional points within the interval derived from the roots of certain orthogonal polynomials. The key advantage of using the Lobatto formula is that it ensures both the differential equation and the boundary conditions are satisfied at carefully chosen collocation points.

In bvp5c, the solver is based on a Lobatto IIIA formula, which is a collocation formula of fourth-order accuracy. By embedding this fourth-order solution into a structure that allows error estimation, the method effectively achieves fifth-order accuracy with adaptive refinement. The Lobatto formula ensures stability and accuracy, making it suitable for stiff problems and complex boundary conditions.

### Collocation method

The **collocation method** is a numerical technique where the solution is approximated by piecewise polynomials, and these polynomials are constrained to satisfy the differential equations at a discrete set of collocation points within each subinterval. In **bvp5c**, the interval is divided into subintervals, and on each subinterval, the solver uses a polynomial of degree four (quartic polynomial) that satisfies the system of ODEs at four Lobatto collocation points.

The collocation conditions can be expressed as:

$$R_{i}=y^{\prime }\left(x_{i}\right)-f\left(x_{i},y_{j}\right)$$

at each collocation point $x_{i}$. These residual equations are solved iteratively using Newton's method.

### Execution of bvp5c

The workflow in **bvp5c** proceeds as follows:

• The solver starts by creating an initial guess for the solution and an initial mesh over the domain.
• On each mesh subinterval, it constructs piecewise polynomial solutions that satisfy the ODE at the Lobatto collocation points.
• It calculates the residual errors and uses these to estimate the global error.
• If the estimated error is above the user-defined tolerance, **bvp5c** refines the mesh automatically, placing more collocation points where the solution changes rapidly.
• This process repeats iteratively until the solution converges to the desired tolerance.

### Advantages of bvp5c over bvp4c

The following table highlights the key difference between bvp5c over bvp4**c.**

**Table:** 

Feature	bvp4c	bvp5c	Benefit of bvp5c
Order of accuracy	4th-order method	5th-order method	Higher accuracy with fewer mesh points
Underlying formula	3-stage Lobatto IIIa formula	4-stage Lobatto IIIa formula	More accurate and stable for stiff problems
Error control	Local error estimate with 4th-order accuracy	Local error estimate with 5th-order accuracy	Improved error estimation and control
Mesh refinement	Adaptive mesh refinement	Adaptive mesh refinement	More efficient and faster convergence
Performance on complex problems	Good	Better for highly nonlinear/stiff BVPs	Handles complex and stiff BVPs more robustly

Initially, the above equations are reformulated to align with the solution framework outlined below.

(23) $$f^{\prime \prime \prime }=\frac{1}{1+\varepsilon }\left(-\left(f+g\right)f^{\prime \prime }+{f^{\prime }}^{2}+ed_{1}{f^{\prime \prime }}^{2}f^{\prime \prime \prime }+Mf^{\prime }\right),$$

(24) $$g^{\prime \prime \prime }=\frac{1}{1+\varepsilon }\left(-\left(f+g\right)g^{\prime \prime }+{g^{\prime }}^{2}+ed_{2}{g^{\prime \prime }}^{2}g^{\prime \prime \prime }+Mg^{\prime }\right),$$

(25) $$\theta^{\prime \prime }=\frac{3Pr}{3+4R}\left(\begin{array}{c} -\left(f+g\right)\theta^{\prime }-N_{b}\theta^{\prime }\phi^{\prime }-N_{t}{\theta^{\prime }}^{2}-Ec_{x}\left({f^{\prime \prime }}^{2}+M{f^{\prime }}^{2}\right)\\ -Ec_{y}\left({g^{\prime \prime }}^{2}+M{g^{\prime }}^{2}\right), \end{array}\right)$$

(26) $$\phi^{\prime \prime }=-LePr\left(f+g\right)\phi^{\prime }-\frac{N_{t}}{N_{b}}\theta^{\prime \prime }.$$

**Fig. 2:**
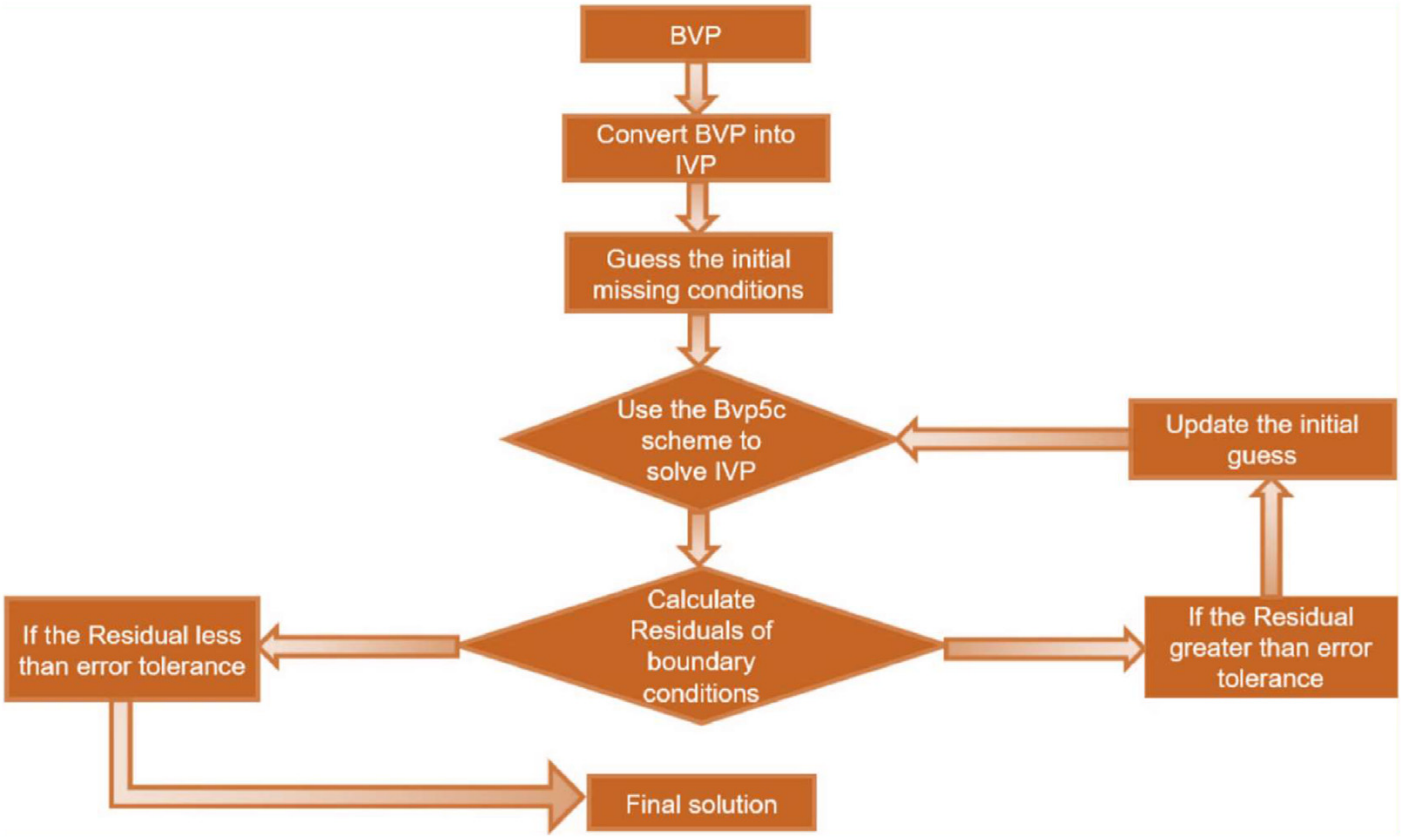
Algorithm of the present scheme.

The system of ordinary differential equations (ODEs) is solved using the renowned numerical approach combining the shooting method and the Four-Stage Lobatto scheme and explore the outcomes of mathematical Eqs. (13)–(16) along with associated conditions outlined in above Eq. (17). Given the complexity of the flow model described by these equations, obtaining analytical solutions is either highly intricate or exceedingly rare with accept able error tolerance is $10^{-6}$. As a result, numerical methods become essential. Our problem in the form of nonlinear system ordinary differential equations, leads to BVP. Consequently, finding a solution is not feasible without employing mathematical solution techniques, such as MATLAB bvp5c (see algorithm Fig. 2). The simulations using MATLAB software facilitate further analysis and endorse the results obtained. Eqs. (13)–(16) are modified into a set of 1st order equations based on the numerical method for ease of computation:

$$\;y_{1}=f;\;y_{2}=f^{\prime };\;y_{3}=f^{\prime \prime };\;y_{4}=g;y_{5}=g^{\prime };\;y_{6}=g^{\prime \prime };y_{7}=\theta ;\;y_{8}=\theta^{\prime };y_{9}=\phi ;y_{10}=\phi^{\prime }.$$

Then the following is obtained:

(27) $$\begin{array}{ccc} {y^{\prime }}_{1} & = & y\left(2\right)\\ {y^{\prime }}_{2} & = & y\left(3\right)\\ {y^{\prime }}_{3} & = & \left(\frac{-1}{\left(1+\epsilon \right)-e*\delta_{1}*y\left(3\right)\wedge 2}\right)*\left(\left(y\left(1\right)+y\left(4\right)\right)*y\left(3\right)-y\left(2\right)\wedge 2-M*y\left(2\right)\right)\\ {y^{\prime }}_{4} & = & y\left(5\right)\\ {y^{\prime }}_{5} & = & y\left(6\right)\\ {y^{\prime }}_{6} & = & \left(\frac{-1}{\left(1+\epsilon \right)-e*\delta_{2}*y\left(6\right)\wedge 2}\right)*\left(\left(y\left(1\right)+y\left(4\right)\right)*y\left(6\right)-y\left(5\right)\wedge 2-M*y\left(5\right)\right)\\ {y^{\prime }}_{7} & = & y\left(8\right)\\ {y^{\prime }}_{8} & = & \left(\frac{-3}{1+4*R}\right)\left(Pr*\left(y\left(1\right)+y\left(4\right)\right)*y\left(8\right)+Pr*Nb*y\left(10\right)*y\left(8\right)+Pr*Nt*y\left(8\right)\wedge 2+E_{cx}*Pr*\left(y\left(3\right)\wedge 2+M*y\left(2\right)\wedge 2\right)\right)\\ {y^{\prime }}_{9} & = & y\left(10\right)\\ {y^{\prime }}_{10} & = & -\left(Le*Pr*(y\left(1\right)+y\left(4\right)\right)*y\left(10\right)+\left(Nt\;/\;Nb\right)*{y^{\prime }}_{8} \end{array}$$

Afterward, the boundary condition may be expressed as:

(28) $$\left\{\;\begin{array}{c} y_{\text{in}1}\left(0\right)=0\\ y_{\text{in}2}\left(0\right)=1\\ \begin{array}{c} y_{\text{in}4}\left(0\right)=0\\ y_{\text{in}5}\left(0\right)=A\\ \begin{array}{c} \;y_{\text{in}9}\left(0\right)=1,\;\\ y_{f}\left(2\right)=0\\ \begin{array}{c} y_{f}\left(5\right)=0\\ y_{f}\left(7\right)=0\\ \begin{array}{c} y_{f}\left(9\right)=0 \end{array} \end{array} \end{array} \end{array} \end{array}\right\}.$$

If the correct values of the variables are assign to the system described by formulas then this system can be numerically integrated automatically using the MATLAB technique bvp5c.

### Method validation

The validation of the Four-Stage Lobatto scheme involves ensuring its reliability, accuracy, and applicability through systematic testing against theoretical, numerical, and real-world benchmarks

### Limitations

While the Four-Stage Lobatto scheme offers significant advantages, it also has inherent limitations:

• Limited applicability to strongly nonlinear problems.
• Computational overhead for complex systems.
• Sensitivity to initial guesses.
• Accuracy is dependent on mesh density.
• Boundary layer and singularities.

## Graphical results and discussion

**Fig. 3:**
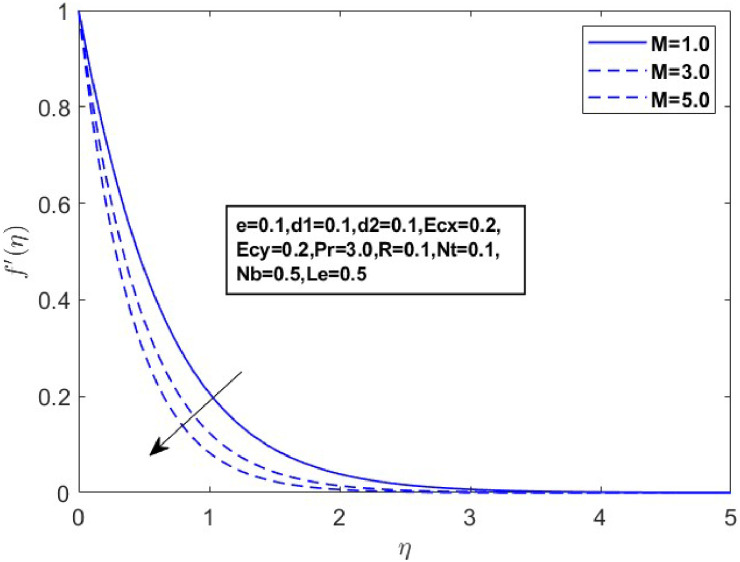
Sketch between Mand $f^{\prime }\left(\eta \right).$

**Fig. 4:**
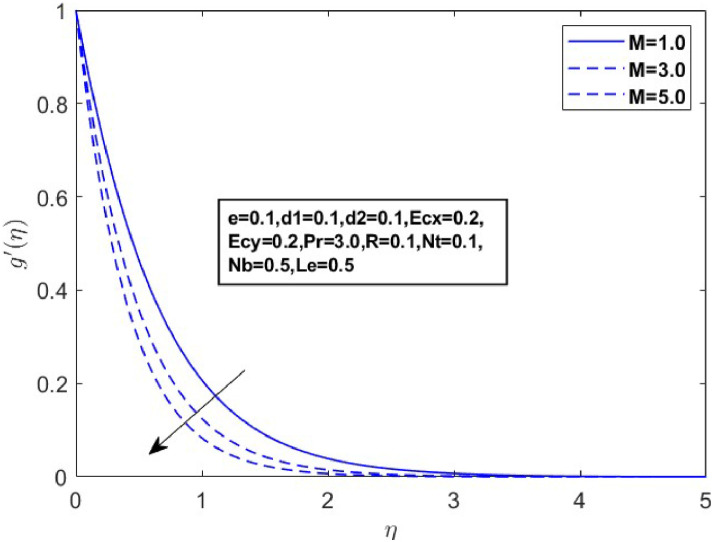
Sketch between Mand $g^{\prime }\left(\eta \right).$

**Fig. 5:**
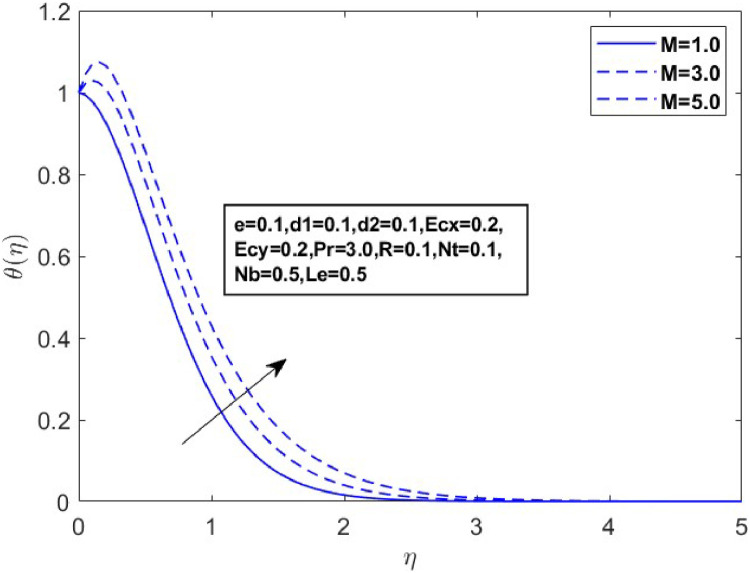
Sketch between Mand θ(η).

**Fig. 6:**
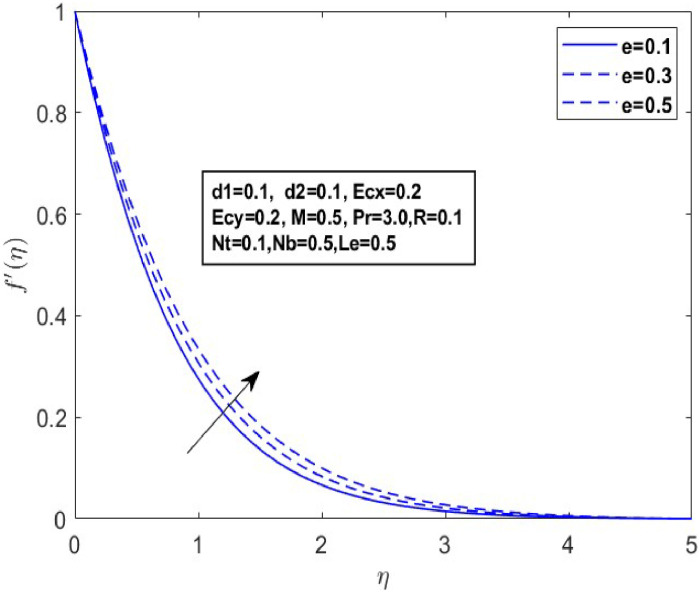
Sketch between e and $f^{\prime }\left(\eta \right).$

**Fig. 7:**
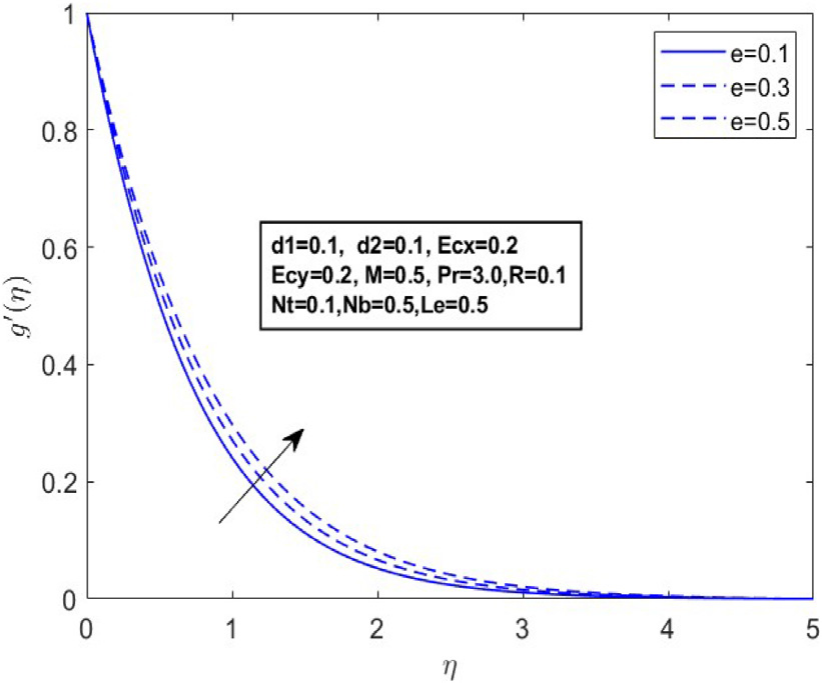
Sketch between eand $g^{\prime }\left(\eta \right)$.

**Fig. 8:**
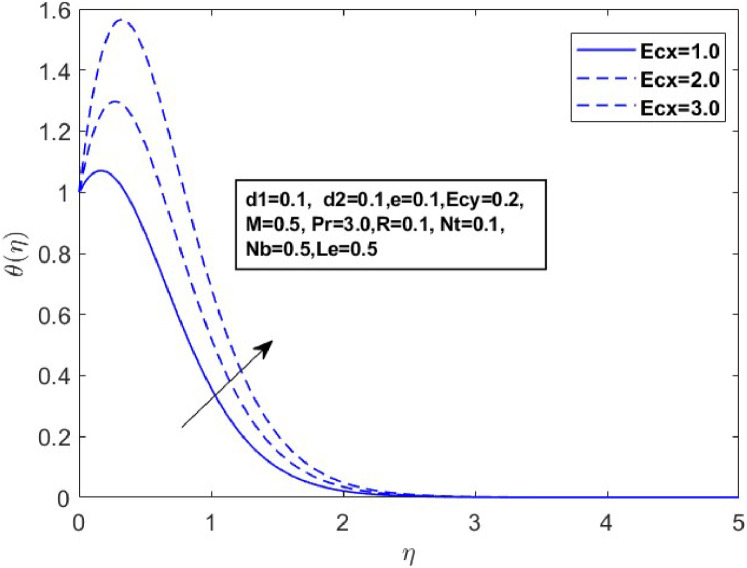
Sketch between $Ec_{x}$ and θ(η).

**Fig. 9:**
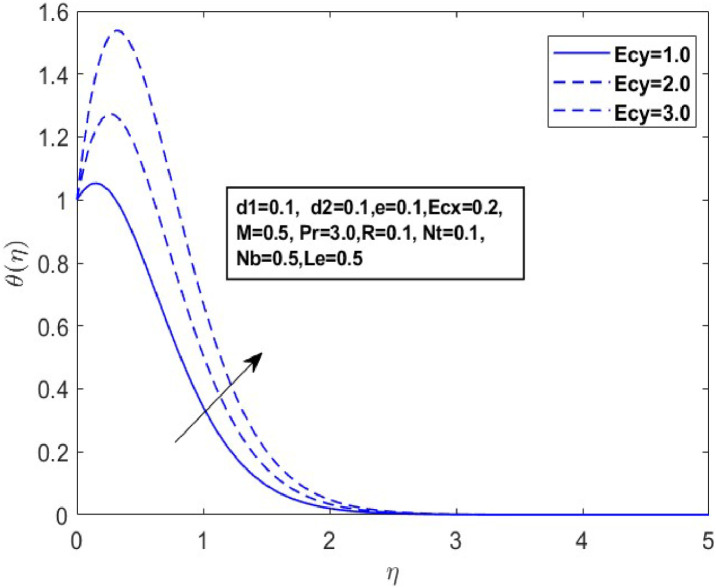
Sketch between $Ec_{y}$ and θ(η).

**Fig. 10:**
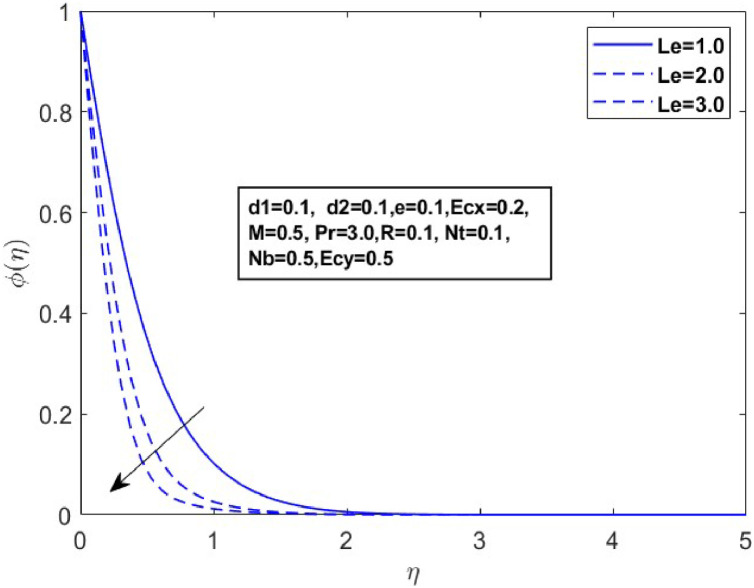
Sketch between $L_{e}$ and ϕ(η).

**Fig. 11:**
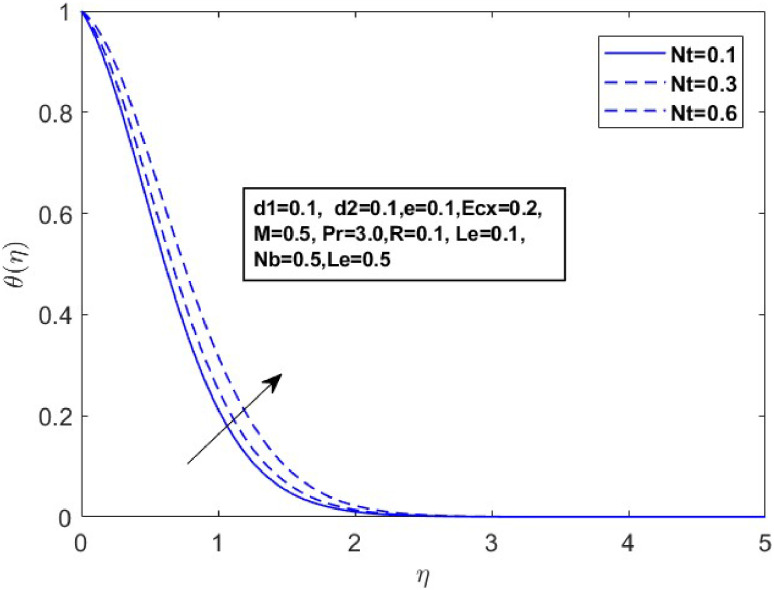
Sketch between $N_{t}$ and θ(η).

**Fig. 12:**
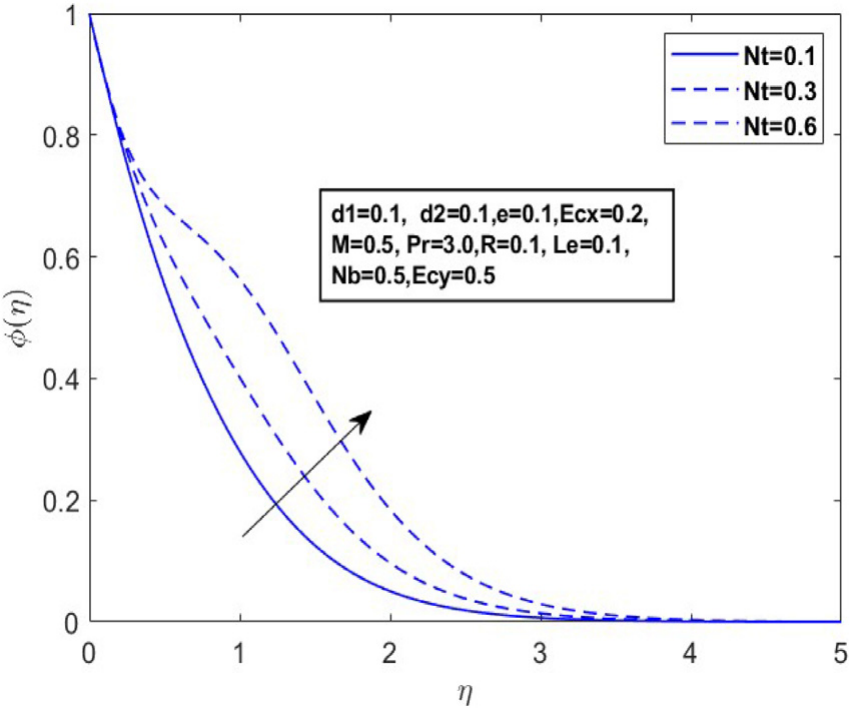
Sketch between $N_{t}$ and ϕ(η).

**Fig. 13:**
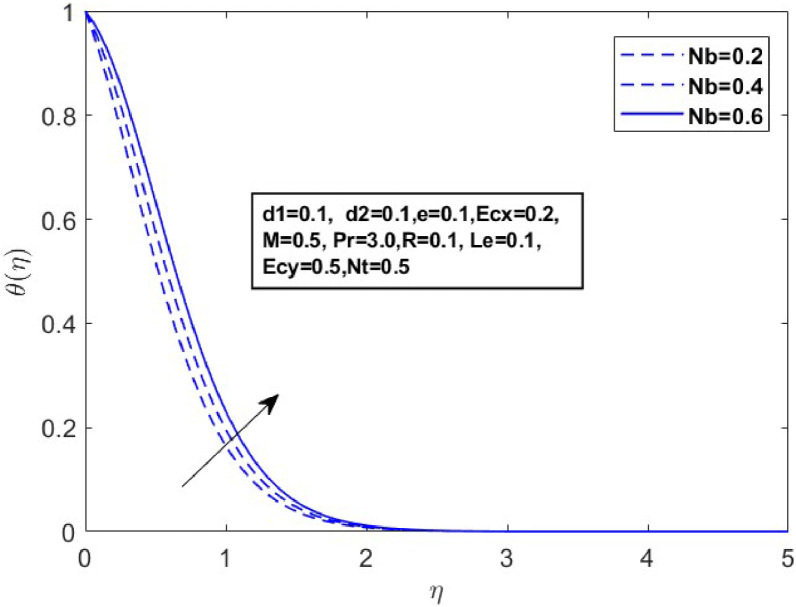
Sketch between $N_{b}$ and θ(η).

**Fig. 14:**
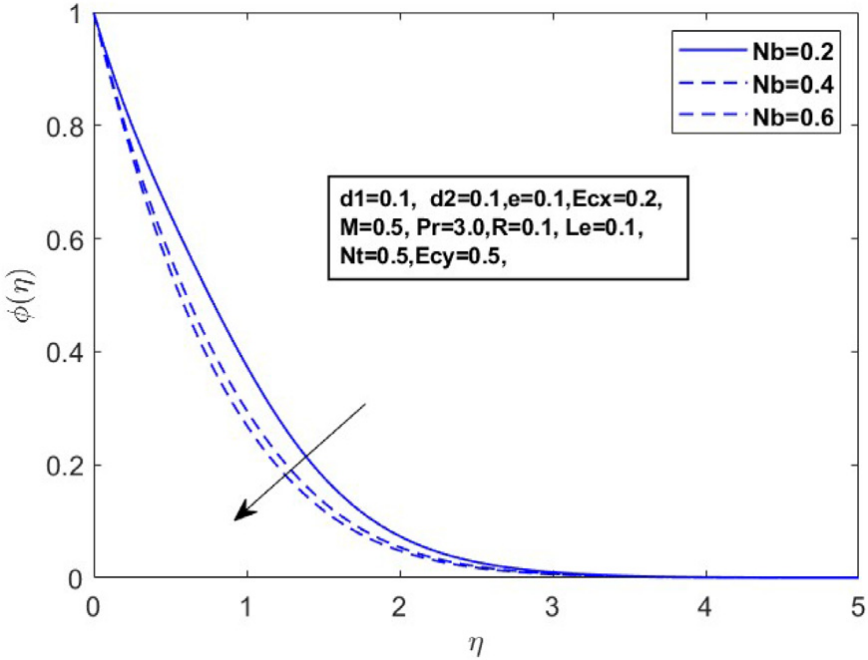
Sketch between $N_{b}$ and ϕ(η).

**Fig. 15:**
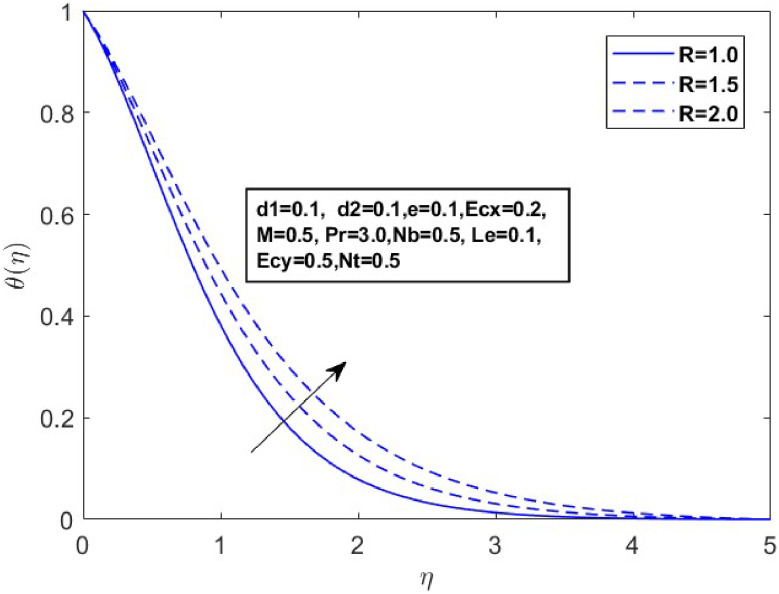
Sketch between R and θ(η).

**Fig. 16:**
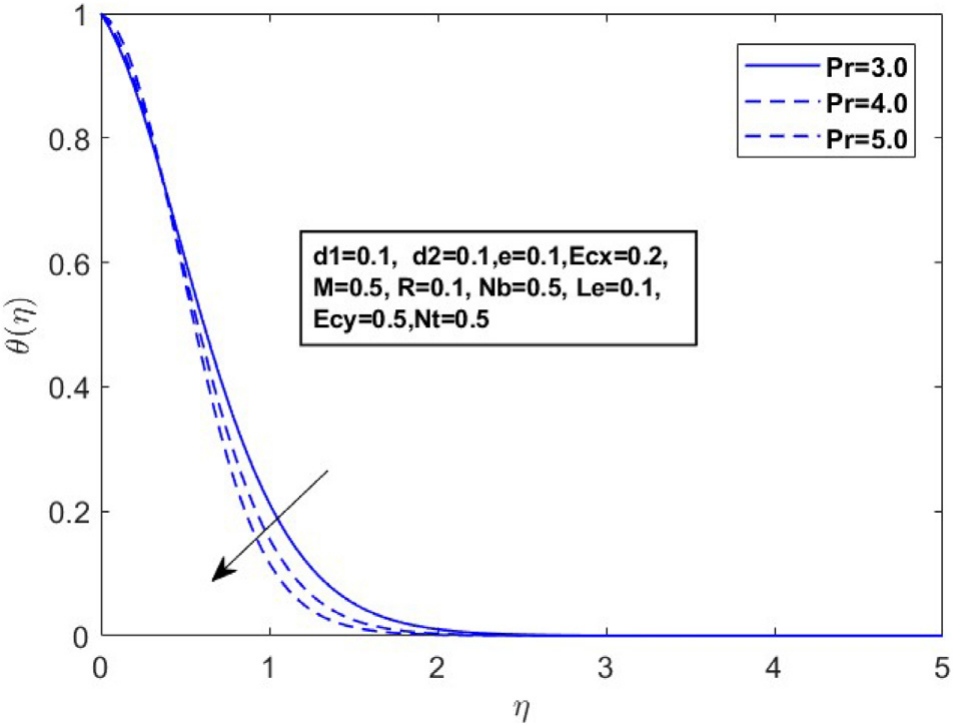
Sketch between $P_{r}$ and θ(η).

**Fig. 17:**
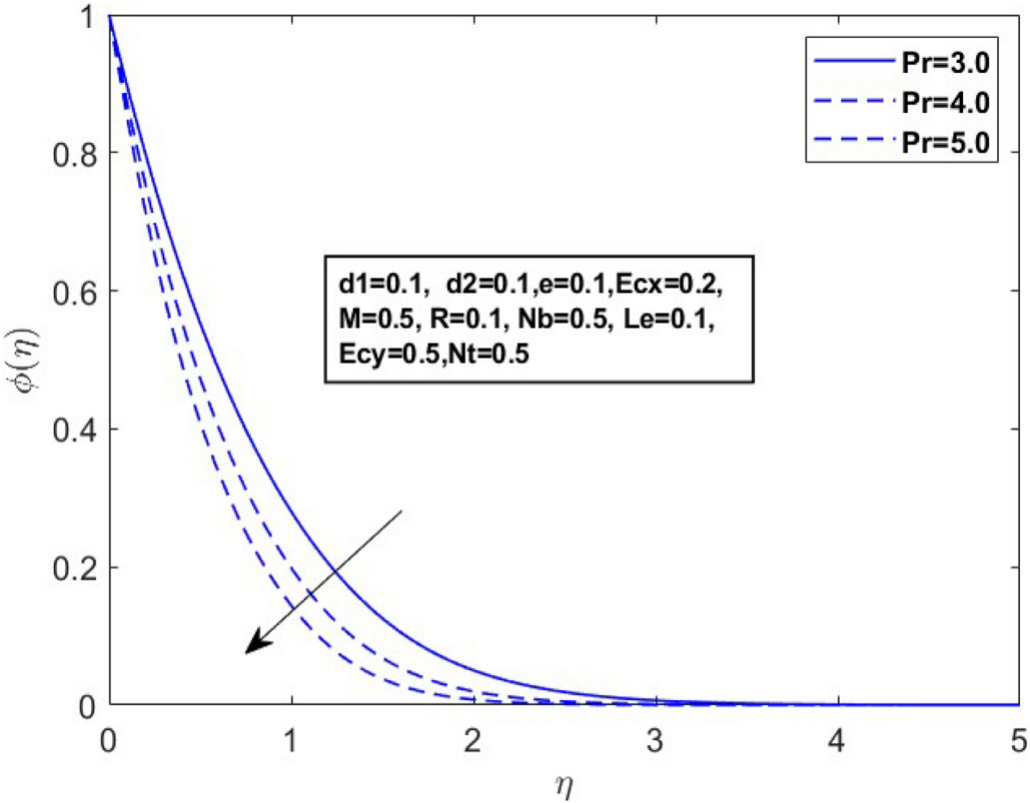
Sketch between $P_{r}$ and ϕ(η).

**Fig. 18:**
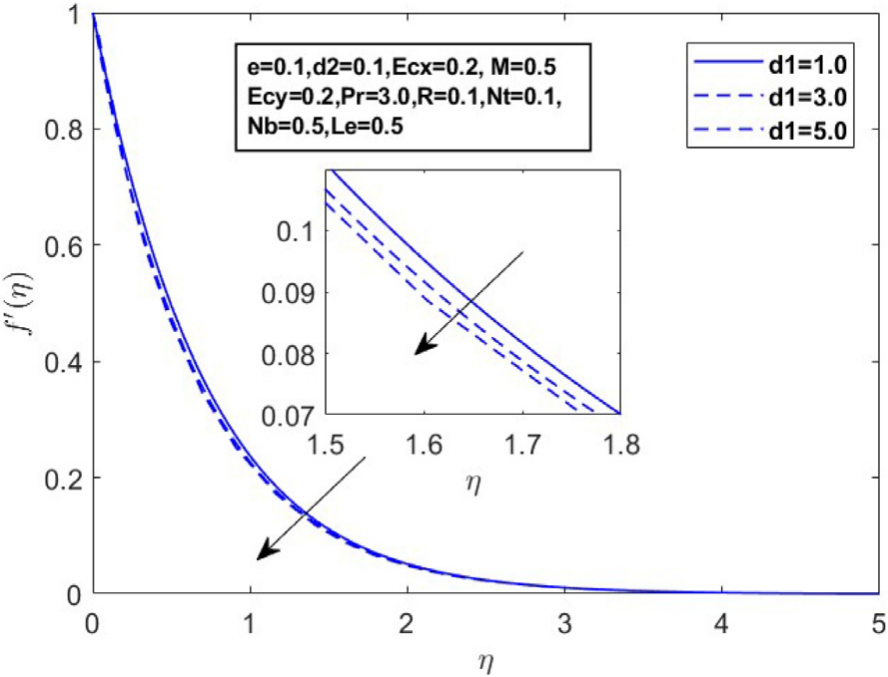
Sketch between $d_{1}$ and $f^{{}^{\prime }\left(\eta \right)}.$

**Fig. 19:**
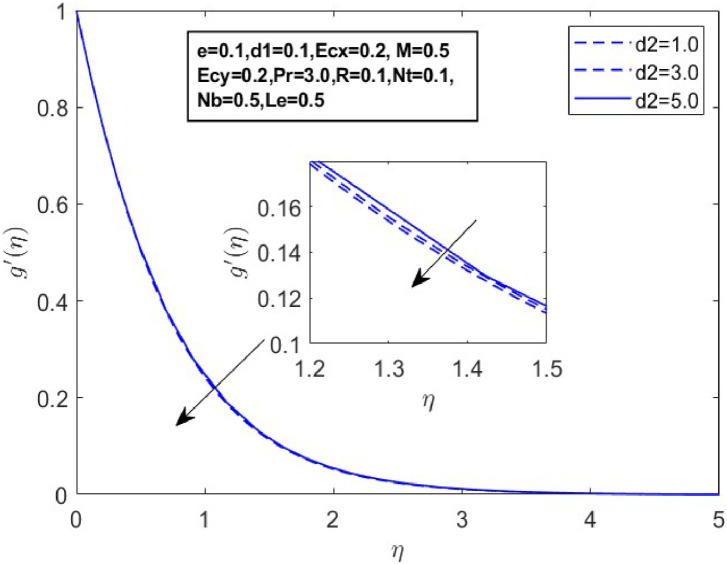
Sketch between $d_{2}$ and $g^{\prime }\left(\eta \right).$

**Fig. 20:**
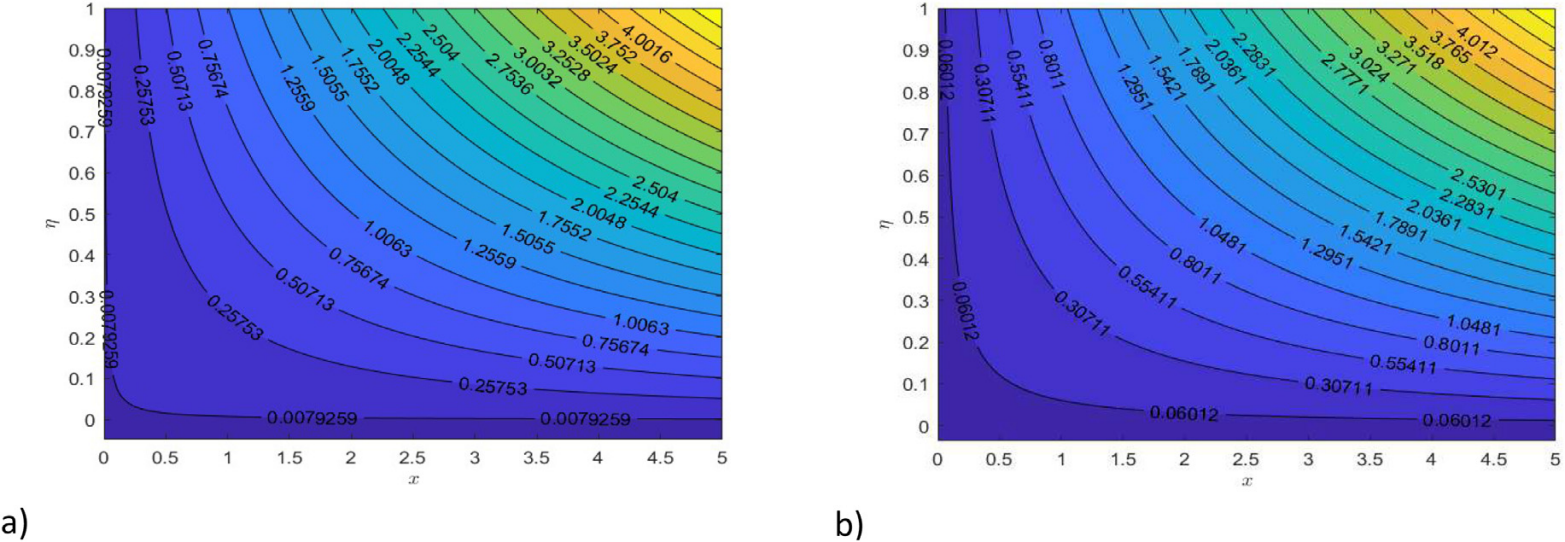
Illustration of streamline for nanofluids, when a) M=0.0, b) M=0.5.

In this section, we derive numerical solutions for the dimensionless boundary value problem. We visually present the profiles of momentum, energy, and concentration across various conditions, including magnetic field strength (M),slip parameters(d1andd2), Eckert numbers ($Ec_{x\;}$and$\;Ec_{y}$), Lewis number (Le), thermophoresis parameter $\left(N_{t}\right)$, and Brownian motion parameter $\left(N_{b}\right)$ are represented graphically in Fig. 1, Fig. 4, Fig. 5, Fig. 6, Fig. 7, Fig. 8, Fig. 9, Fig. 10, Fig. 11, Fig. 12, Fig. 13, Fig. 14, Fig. 15, Fig. 16, Fig. 17, Fig. 18, Fig. 19, Fig. 20. These mathematical solutions are meticulously calculated to account for the diverse physical factors$e=0.1,\;d1=0.1,\;d2=0.1,\;Ec_{x}=0.2,\;\;Ec_{y}=0.2,\;M=0.5,\;Nt=0.1,\;Nb=0.5,\;Le=0.5$. Table 1 validates our proposed method, showing good agreement with the published data. Table 2, Table 3 present the values of the skin friction coefficient and Nusselt number for various relevant parameters, along with their corresponding trends and behaviors. Fig. 3, Fig. 4, Fig. 5 shows contours of temperature θ(η), velocity $f^{\prime }\left(\eta \right)\;$& $g^{\prime }\left(\eta \right)$, with magnetic field (M). The Lorentz force is an opposing force that arises when a magnetic field interacts with a conducting fluid. This results in a decrease in the derivatives of $f^{\prime }\left(\eta \right)\;and\;g^{\prime }\left(\eta \right)$. From a physical perspective, the Lorentz force acts as a resistance to the fluid's motion, arising due to the interaction between the magnetic field and the electrically conducting fluid. As the Hartmann number increases, this force intensifies, effectively suppressing all components of the fluid velocity. By enhancing the magnetic number M a slight enhancement in the temperature profile is observed in correlation with this process. This depicts how the magnetic field counteracts transportation events. It is crucial to admit that the elevated resistances in the fluid components result in the heating of the flow field, leading to a vertical increase in the magnetic field. This feature exhibits an inverse relationship with the dynamic viscosity of a non-Newtonian fluid.

**Table 1:** A comparative analysis to investigate the outcomes with shooting of $f^{\prime \prime }$(0) and $g^{\prime \prime }$(0).

Parameters		[34]	[35]	Shooting Technique	Present results	[34]	[35]	Shooting technique	Presents results
M	λ	$-f^{\prime \prime }\left(0\right)$	$-f^{\prime \prime }\left(0\right)$	$-f^{\prime \prime }\left(0\right)$	$-f^{\prime \prime }\left(0\right)$	$-g^{\prime \prime }\left(0\right)$	$-g^{\prime \prime }\left(0\right)$	$-g^{\prime \prime }\left(0\right)$	$-g^{\prime \prime }\left(0\right)$
0	0	1	1	1	1	0	0	0	0
1	0	1.414214	1.414214	1.4142121	1.414213	0	0	0	0
0	0.5	1.093096	1.093095	1.0931052	1.093097	0.465205	0.465205	0.4652135	0.465206
1	0.5	1.476770	1.476771	1.4767702	1.476771	0.679809	0.679809	0.6798091	0.679808
0	1	1.173722	1.173722	1.1737233	1.173722	1.173721	1.173722	1.1737232	1.173722
1	1	1.535711	–	1.5357101	1.535711	1.535710	–	1.5357101	1.535711

**Table 2:** Derived measurement of skin friction and Nusselt number.

M	d1	d2	e	$-f{\;}^{\prime \prime }\left(0\right))$	$-g{}^{\prime \prime }\left(0\right)$
0.1				1.1612	1.1611
0.2				1.9913	1.9912
0.3				1.2360	1.2360
0.4				1.2720	1.2720
	0.1			1.1613	1.1613
	0.2			1.1647	1.1612
	0.3			1.1682	1.1611
	0.4			1.1718	1.1610
		0.1		1.1613	1.1613
		0.2		1.1612	1.1647
		0.3		1.1610	1.1682
		0.4		1.1609	1.1717
			0.2	1.1141	1.1141
			0.4	1.0340	1.0340
			0.6	0.9686	0.9686
			0.8	0.9140	0.9040

**Table 3:** Derived measurement of skin friction and Nusselt number.

R	$N_{b}$	$N_{t}$	$E_{cx}$	$E_{cy}$	Le	$-\theta {}^{\prime }\left(0\right))$	$-\phi {}^{\prime }\left(0\right))$
0.1						0.4143	1.0994
0.2						0.4287	1.0951
0.3						0.4379	1.0917
0.4						0.4434	1.0891
	0.5					0.4144	1.0995
	0.6					0.3297	1.1129
	0.7					0.2541	1.1213
	0.8					0.1873	1.1266
		0.1				0.4144	1.0995
		0.2				0.3646	1.009
		0.3				0.3189	1.1147
		0.4				0.2770	1.1389
			0.2			0.4144	1.0995
			0.3			0.3137	1.1166
			0.4			0.2129	1.1339
			0.5			0.1119	1.1511
				0.2		0.4144	1.0995
				0.3		0.3137	1.1166
				0.4		0.2129	1.339
				0.5		0.1119	1.1511
					0.5	0.4142	1.0995
					0.6	0.3823	1.2428
					0.7	0.3578	1.3732
					0.8	0.338	1.4936

A drop in flow resistance brought on by a rise in e causes the fluid velocity to increase. Fig. 6, Fig. 7 and 7 illustrate how the non-Newtonian fluid parameter e affects the distributions of $f^{\prime }\left(\eta \right),\;\text{and}\;g^{\prime }\left(\eta \right)$. The graph shows that dispersals near the boundary layer increase as the values of erise. Figs. 8 and 9 illustrate the temperature profiles for various values of the Eckert number$\;(E_{cx\;},\;E_{cy})$. These data indicate that temperature (θ(η)) becomes wider as $E_{cx\;}$and $E_{cy}$ improve and closer to the boundary layer within the range of 1≤η≤3. From a physical standpoint, an increase in the Eckert number leads to a rise in temperature. This is because the Eckert number represents the ratio of kinetic energy to enthalpy; higher values indicate that viscous dissipation significantly converts the fluid kinetic energy into thermal energy, thereby elevating the temperature.

Fig. 10 displays the dynamics of the temperature and concentration curves with the Lewis number (Le) parameter. The region labeled as Le in the boundary layer indicates the proportionate influence of heat diffusion on species diffusion, resulting in a decrease in the Lewis number as a consequence of the decline in a species boundary layer. From a physical perspective, an increase in the Lewis number (Le), which is the ratio of thermal diffusivity to mass diffusivity, results in a decrease in the concentration profile. This occurs because a higher Lewis number indicates that mass diffusivity is relatively lower compared to thermal diffusivity, leading to slower mass transport and a thinner concentration boundary layer. The temperature and concentration boundary layer equations in Figs. 11 and 12 contain parameters associated with Brownian motion and thermophoresis. $N_{t\;}$are impacted by both temperature and concentration and they have a major effect on the diffusion of heat and concentration of nanoparticles in the boundary layer. In the next investigation, we will examine the impact of $N_{b}$ on θ(η)andϕ(η) profiles using Figs. 13 and 14. It has been noted that the temperature of the fluid rose significantly as the values of $N_{b}$ increased. Conversely, the proportion of nanoparticles in the mixture increases as the total number of particles ($N_{b\;}$) improves but the opposite structure is found when the number of nanoparticles ($N_{b}$) fluctuates. This is because the random movement of nanoparticles gets stronger as the Brownian motion parameter increases, leading to an increase in fluid temperature and a decrease in nanoparticle diffusion.

Fig. 15 illustrates how the profiles of θ(η)within the boundary layer shift as the radiation parameter R changes, under both steady and turbulent flow conditions. As R increases, there is a marked expansion in both the temperature field and the thermal boundary layer thickness. A higher radiation parameter promotes more transfer of heat, thereby thickening the thermal boundary layer. Additionally, R tends to reduce the volume fraction close to the surface, whereas the nanoparticle concentration increases further away from the sheet. From a physical perspective, an increase in the radiation parameter enhances the transfer of thermal energy within the fluid, leading to a rise in the temperature profile. This is because the radiation parameter represents the relative contribution of radiative heat transfer to conduction; higher values signify stronger radiative effects, which intensify the thermal energy distribution across the medium. Figs. 16 and 17 explore how the Prandtl number (Pr) affects temperature and concentration distributions. Fig. 16 illustrates the interplay between temperature and concentration as influenced by the Prandtl number, while Fig. 17 specifically highlights concentration changes. These figures reveal that higher Prandtl numbers, which indicate reduced thermal diffusivity, lead to decreased fluid temperature and concentration. As the Prandtl number rises, thermal diffusivity decreases, causing a drop in both temperature and concentration and contributing to the formation of a thermal boundary layer. The influence of the Powell-Eyring fluid parameters, d1, and d2 on the velocity profiles is explained in Figs. 18 and 19. As d1andd2 grow the velocity profiles become thinner and the momentum boundary layer thickness gets thicker. This is thus because Powell-Eyring fluids which decrease viscosity as the shear rate increases are shear thinning fluids. Visualization of streamline against magnetic parameter portrayed in the Fig. 20 for two cases when M=0.0 & M=0.5.

## Conclusion

This study delves into the exploration of three-dimensional Eyring-Powell flow in a radiative magnetic nanofluid over a stretchable surface, incorporating convective boundary conditions. The analysis seamlessly integrates the impact of thermophoresis and Brownian motion. The governing equations are simplified and solved using MATLAB built-in computational scheme. The main findings of this investigation are outlined as follows:

• Fluid velocity decreases as the magnetic field strength increases, while it increases with the Eyring-Powell fluid parameter.
• Fluid heat rises with a stronger magnetic field and higher Biot number, but decreases with a higher slip parameter.
• Skin friction increases with higher thermophorsis parameter and Biot number.
• Nusselt number improves with Lewis number initially but decreases at higher Lewis numbers.
• Enhancement in noted in heat transfer rate for radiation.

## Ethical approval

All procedures performed in the studies comply with ethical standards.

## Research involving humans and animals statement

None.

## CRediT authorship contribution statement

**Aaqib Majeed:** Writing – review & editing, Writing – original draft, Visualization, Validation, Supervision, Software, Resources, Project administration, Methodology, Investigation, Formal analysis, Conceptualization. **Parvez Ali:** Writing – review & editing, Methodology, Software, Formal analysis, Resources. **Marouan Kouki:** Investigation, Methodology, Software, Validation, Visualization, Supervision, Writing – review & editing, Data curation, Conceptualization.

## Declaration of competing interest

Authors declares that they have no potential conflict of interest

## Data Availability

Data will be made available on request.
